# Clinical Characteristics of Adrenal Hemangioma

**DOI:** 10.1210/jendso/bvae041

**Published:** 2024-03-20

**Authors:** Yuzhi Zuo, Zhen Liang, Shengmin Yang, Boju Pan, Sihang Cheng, Zhien Zhou, Tianrui Feng, Weigang Yan, Xingcheng Wu

**Affiliations:** Department of Urology, Peking Union Medical College Hospital, Peking Union Medical College, Chinese Academy of Medical Sciences, Beijing 100730, China; Department of Urology, Peking Union Medical College Hospital, Peking Union Medical College, Chinese Academy of Medical Sciences, Beijing 100730, China; Department of Urology, Peking Union Medical College Hospital, Peking Union Medical College, Chinese Academy of Medical Sciences, Beijing 100730, China; Department of Pathology, Peking Union Medical College Hospital, Peking Union Medical College, Chinese Academy of Medical Sciences, Beijing 100730, China; Department of Radiology, Peking Union Medical College Hospital, Peking Union Medical College, Chinese Academy of Medical Sciences, Beijing 100730, China; Department of Urology, Peking Union Medical College Hospital, Peking Union Medical College, Chinese Academy of Medical Sciences, Beijing 100730, China; Department of Urology, Peking Union Medical College Hospital, Peking Union Medical College, Chinese Academy of Medical Sciences, Beijing 100730, China; Department of Urology, Peking Union Medical College Hospital, Peking Union Medical College, Chinese Academy of Medical Sciences, Beijing 100730, China; Department of Urology, Peking Union Medical College Hospital, Peking Union Medical College, Chinese Academy of Medical Sciences, Beijing 100730, China

**Keywords:** adrenal hemangioma, clinical characteristics, enhanced CT, hormone examinations, surgical management

## Abstract

**Objective:**

Adrenal hemangioma (AH) is a rare, benign adrenal tumor often detected incidentally by imaging. This retrospective study aimed to investigate the clinical characteristics of AH, including clinical and diagnostic imaging features, to improve the recognition and understanding of AH.

**Methods:**

We retrospectively analyzed the medical records of patients diagnosed with AH at Peking Union Medical College Hospital between 2008 and 2022. Clinical manifestations, adrenal hormone levels, imaging findings, treatment approaches, and pathological results were collected and analyzed.

**Results:**

Of the 7140 adrenal tumor patients, 40 (0.56%) had AH confirmed postoperatively. The mean age at diagnosis was 53.9 years, with a female predominance. Most (70%) were asymptomatic and diagnosed incidentally. Misdiagnosis before surgery was common, most frequently as pheochromocytoma. Imaging characteristics, especially enhanced computed tomography, revealed distinct features based on tumor size. Surgery was the main treatment, with laparoscopic adrenalectomy preferred.

**Conclusion:**

This study elucidates the clinical characteristics of AH, including demographics, diagnostic challenges, and imaging features. AH often presents incidentally and is frequently misdiagnosed preoperatively. Recognizing distinct imaging characteristics and appropriate surgical management can enable accurate diagnosis and optimal treatment.

Hemangioma is a well-defined tumor composed of vascular endothelium, which is most common in the head, neck, and liver. Adrenal hemangioma (AH) is a rare, benign tumor usually found incidentally on imaging. Most are cavernous types; capillary and other types are rare [1]. Since the first reported case in 1955 [2], approximately 100 cases have been described, but most are case reports or small case series [1, 3, 4], limiting comprehensive understanding of AH clinical characteristics. Preoperative diagnosis is challenging, with pheochromocytoma the most frequent misdiagnosis, resulting in unnecessary preoperative medication. The majority of patients are asymptomatic, and most adrenal hemangiomas have no endocrine function. However, some patients may experience shock from rupture hemorrhage or adrenal hormone abnormalities, complicating diagnosis and treatment. Recently, the incidence of AH has increased due to the evolution of cross-sectional imaging including computed tomography (CT) and magnetic resonance imaging (MRI). In the present study, we summarized the clinical and imaging features of AH patients diagnosed at our hospital from 2008 to 2022, aimed at improving recognition and understanding of this tumor.

## Materials and Methods

We retrospectively analyzed records of adrenal tumor patients admitted to the Urology Department of Peking Union Medical College Hospital from 2008 to 2022. Patients without enhanced CT images or who underwent surgeries in other hospitals were excluded. Clinical manifestations, adrenal hormone levels, imaging findings, treatment approaches, and pathological results were collected and analyzed. This study was approved by the Ethics Committee of Peking Union Medical College Hospital. Serum potassium and adrenal hormones examinations including plasma ACTH, plasma cortisol, 24-hour urinary free cortisol (UFC), plasma renin activity and aldosterone, 3-metanephrine, 3-normetanephrine, dehydroepiandrosterone, and 24-hour urinary catecholamines (epinephrine, norepinephrine, and dopamine) were analyzed. Imaging tests included ultrasonography and enhanced CT. Maximal tumor diameter, echogenicity, and contrast enhancement patterns were analyzed and categorized as none (<5 HU change), mild (5-20 HU), moderate (20-50 HU), or marked (>50 HU) based on attenuation value difference between unenhanced and arterial phase.

Surgical resections were performed through open or laparoscopic surgery based on the preoperative diagnosis, tumor size, and surgeon experience. Operation time, estimated blood loss, and complications were evaluated. Pathological analysis confirmed the diagnosis.

## Results

### Clinical Manifestations of AH Patients

Of the 7140 adrenal tumor patients who underwent adrenal surgeries at our hospital from 2008 to 2022, 40 (0.56%) were confirmed to have AH based on postoperative pathology. Thirty-six of them had isolated AH, and 4 of them had adrenal collisions tumors with AH and adrenal adenoma/pheochromocytoma. The mean age at diagnosis was 53.9 years (range 13-76 years), with a 5:3 female predominance. Most patients (70%) were asymptomatic and diagnosed incidentally during health examinations. Only 2 patients (5%) presented with back or abdominal pain. Ten patients (25%) were diagnosed due to difficult-to-control hypertension or hypertension-related complications. This included 2 patients of new-onset uncontrolled hypertension, 6 patients of hypertension with headaches or palpitations, and 2 patients of hypertension with hypokalemia. Well-controlled hypertension was the most common comorbidity, present in 18 patients (45%). Only 1 patient was clinically diagnosed with AH before surgery. Pheochromocytoma was the most frequent preoperative diagnosis, accounting for 17 cases (42.5%). Other preoperative diagnoses included adrenal adenoma with unclear function in 6 cases (15%), nonfunctional adenoma in 4 cases (10%), adrenal cortical carcinoma in 3 cases (7.5%), aldosterone-producing adenoma in 3 cases (7.5%), adrenal cysts in 3 cases (7.5%), adrenal hemangioma in 1 case (2.5%), adrenal tuberculosis in 1 case (2.5%), ganglioneuroma in 1 case (2.5%), and metastatic tumor in 1 case (2.5%) (Tables 1 and 2).

**Table 1.** Demographic and clinical characteristics of the 40 patients with adrenal hemangioma

Demographic and clinical characteristics	Value (n = 40)
Age at diagnosis (mean ± SD, years)	53.9 ± 13.9
Distribution of age at diagnosis (%)	
<18	1 (2.5)
18-44	8 (20)
45-64	23 (57.5)
65-74	7 (17.5)
≥75	1 (2.5)
Sex (%)	
Male	15 (37.5)
Female	25 (62.5)
Clinical presentations (%)	
Incidental	28 (70)
Back/abdominal discomfort	2 (5)
Hypertension with complications (%)	
Uncontrolled hypertension	2 (5)
Hypertension and headache	3 (7.5)
Hypertension and palpitation	3 (7.5)
Hypertension and hypokalemia	2 (5)
Comorbidities and post history (%)	
Hypertension	18 (45)
Diabetes	8 (20)
Cerebral hemorrhage	4 (10)
Clinical diagnosis (%)	
Pheochromocytoma	17 (42.5)
Adrenal adenoma with unclear function^a^	6 (15)
Nonfunctional adrenal adenoma^b^	4 (10)
Adrenal carcinoma	3 (7.5)
Aldosterone-producing adenoma	3 (7.5)
Adrenal cyst	3 (7.5)
Adrenal hemangioma	1 (2.5)
Adrenal tuberculosis	1 (2.5)
Ganglioneuroma	1 (2.5)
Metastatic tumor	1 (2.5)
MIBG/somatostatin receptor scintigraphy results (%)	
MIBG positive	2/13 (15.4)
Somatostatin receptor scintigraphy positive	4/26 (15.4)
Both positive	1/11 (9)

Abbreviations: MIBG, metaiodoenzylguanidine.

**Table 2.** Results of adrenal endocrine hormone tests

No.	MIBG	Somatostatin receptor scintigraphy	Aldosterone (6.5-29.6 ng/dL)	PRA (0.93-6.56 ng/mL/h)	24h UFC (12.3-103.5 μg/24h)	ACTH (0-46 pg/mL)	F (4-22.3 μg/dL)	MN (0-0.5 nmol/L)	NMN (0-0.9 nmol/L)	DS (12-133 μg/dL)	24h urinary CA		
											DA (0-459.9 μg/24h)	E (0-11 μg/24h)	NE (0-76.9 μg/24h)
1	/	N	16.61	2.87	69.4	7.7	6.8	/	/	/	Normal	Normal	Normal
2	/	N	Normal	Normal	Normal	Normal	Normal	Normal	Normal	/	Normal	Normal	Normal
3	N	N	13.14	/	/	<5	1.1	Normal	Normal	/	/	/	/
4	/	/	14.18	0.17	25.8	16.5	13.5	Normal	Normal	118	Normal	Normal	Normal
5	N	/	/	0.02	18.8	14.5	/	Normal	Normal	/	Normal	Normal	Normal
6	/	/	/	/	181	Normal	Normal	Normal	Normal	/	/	/	/
7	/	N	10.439	0.01	14.7	32.5	7.7	Normal	Normal	159.6	/	/	/
8	/	N	Normal	0.01	Normal	17	Normal	/	/	/	Normal	Normal	Normal
9	N	N	/	0.24	82.4	/	22.5	/	/	/	Normal	Normal	Normal
10	/	N	Normal	0.01	Normal	Normal	Normal	/	/	/	Normal	Normal	Normal
11	/	/	18.7	0.01	79.8	5	/	Normal	Normal	99	340.02 (120.93-330.59)^a^	Normal	Normal
12	/	/	14.04	0.01	51	26	26.5	/	/	/	Normal	Normal	Normal
13	/	/	Normal	Normal	Normal	Normal	Normal	/	/	/	/	/	/
14	/	/	17.08	0.1	Normal	54	/	/	/	91.7	/	/	/
15	/	/	15.78	0.01	60.04	21.4	17.81	/	/	292.4	Normal	Normal	Normal
16	/	N	/	/	69	31.3	19	/	/	95	Normal	Normal	Normal
17	N	N	13.04	2.51	54.12	39.2	/	/	/	/	Normal	Normal	50.23 (16.69-40.65)^a^
18	N	/	28.68	1.13	93.72	58.5-246	7.48	/	/	611.3 (38-313)^a^	Normal	Normal	Normal
19	N	N	13.2	0.01	/	19.4	12.62	/	/	/	Normal	Normal	Normal
20	N	N	15.13	0.16	77	36.1	13.54	/	/	/	Normal	Normal	Normal
21	N	P	13.71	1.45	49.61	37.3	13.89	/	/	/	Normal	Normal	Normal
22	N	N	15.03	2.65	69.5	6.3	/	/	/	/	Normal	Normal	Normal
23	P	N	11.69	0.56	44.16	25.2	/	/	/	/	Normal	Normal	Normal
24	/	/	Normal	Normal	/	/	/	/	/	/	Normal	Normal	Normal
25	/	N	24.06	0.18	42	25	/	/	/	/	Normal	Normal	Normal
26	/	N	12.16	0.67	/	14.1	14.8	/	/	/	Normal	Normal	Normal
27	N	N	9.84	0.01	56	15.6	7.79	/	/	/	Normal	Normal	Normal
28	N	N	14.64	0.42	88.1	21.1	19.93	/	/	/	Normal	Normal	Normal
29	/	N	17.94	0.62	63.18	21.5	17.19	/	/	/	Normal	Normal	Normal
30	/	/	Normal	Normal	Normal	Normal	Normal	/	/	/	Normal	Normal	Normal
31	/	P	11.18	0.62	257.9	43	32.71	/	/	/	350.82	Normal	Normal
32	/	/	13.84	0.88	55.7	5-59	13.34-17.66	/	/	/	/	/	/
33	/	N	/	/	92.9	/	/	/	/	/	Normal	Normal	Normal
34	/	N	16.32	0.05	/	21.7	7.33	/	/	151.2	Normal	Normal	Normal
35	/	/	19.06^b^	0.1	/	27.2	17.37	/	/	/	Normal	Normal	Normal
36	/	N	/	/	/	13.8	12.75	/	/	/	/	/	/
37	N	/	10.45	0.23	48.2	6.6	/	Normal	Normal	/	487.9	Normal	Normal
38	/	P	/	/	53.6	7.1	/	/	/	/	Normal	Normal	Normal
39	/	N	24.93	0.35	138.5	/	/	/	/	/	/	/	/
40	P	P	18.48	0.95	/	32.7	23.9	0.09	18.4-28.19	134	1005.8-1194	5.2	838.9-857.6

Abbreviations: DA, dopamine; DS, dehydroepiandrosterone; E, epinephrine; MIBG, metaiodoenzylguanidine; MN, metanephrine, NE, norepinephrine; NMN, 3-normetanephrine; PRA, plasma rennin activity; UFC, urinary-free cortisol.

Sixteen patients were misdiagnosed as pheochromocytoma. Among them, 2 patients presented with paroxysmal hypertension accompanied by headache/palpitations and profuse sweating and had positive results of somatostatin receptor scintigraphy. One patient (no. 23) had a positive result of metaiodoenzylguanidine (MIBG) despite the absence of typical clinical manifestations. In addition, the other 13 patients were suspected of having pheochromocytoma because enhanced CT showed obvious enhancement of adrenal masses. No significant hypercatecholaminemia was detected among all included patients.

### Imaging Features of AH Patients

The mean tumor diameter of AH was 4.07 cm (range 1.0-10.9 cm) on CT. Most were unilateral, with a right-sided predominance (23 right vs 16 left). One patient (no. 18) had bilateral adrenal masses on imaging but only underwent a right adrenalectomy, leaving the left pathology unknown.

**Figure 1.**
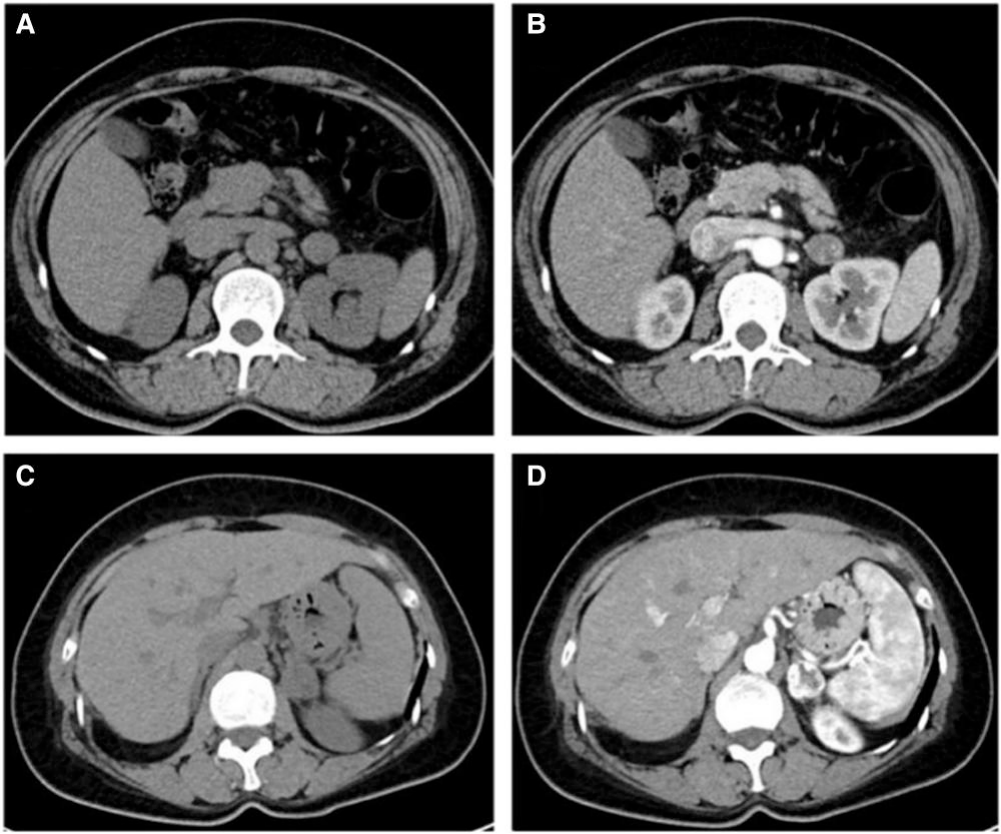
Contrast-enhanced computed tomography demonstrating different enhancement patterns of different size tumors. (A, B) shows mild enhancement of a 1.8 cm tumor and (C, D) shows marked enhancement of a 2.3 cm tumor in the arterial phase.

**Figure 2.**
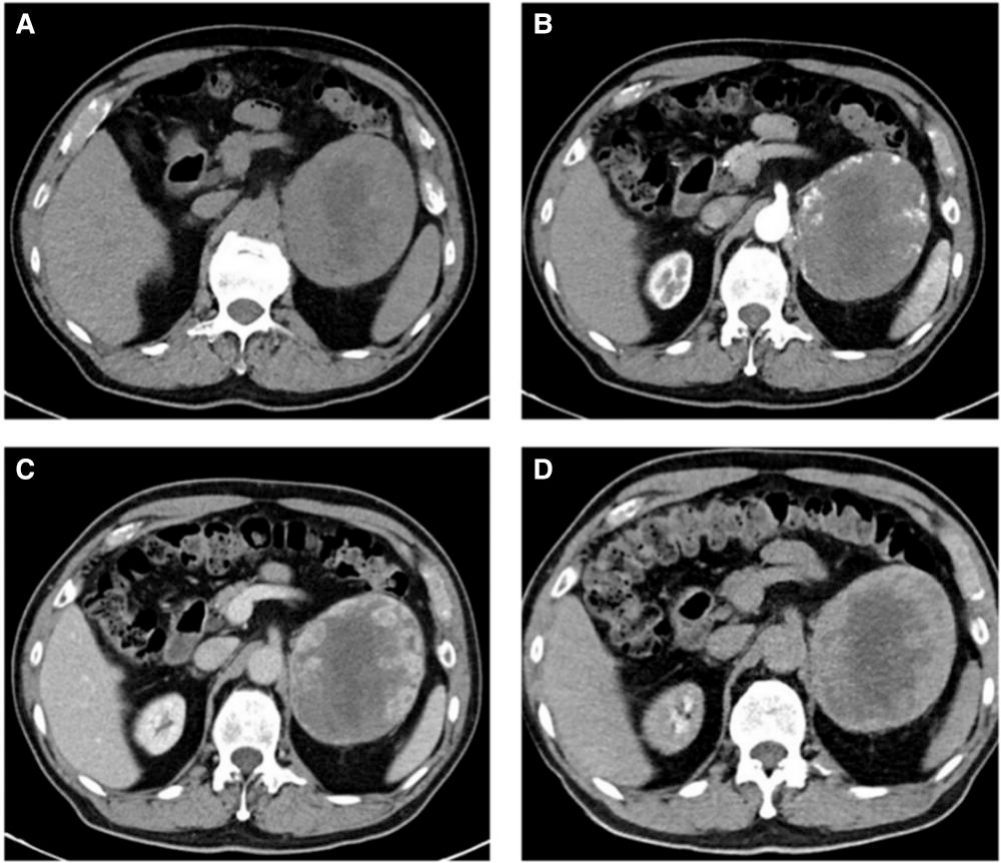
Contrast-enhanced computed tomography demonstrating typical centripetal progressive enhancement and central necrosis. (A) Unenhanced phase; (B) arterial phase demonstrating peripheral nodular enhancement; (C) venous phase showing centripetal filling; (D) delayed phase exhibiting progressive central enhancement.

On ultrasonography, the appearances lacked specificity, with variable echogenicity including hypoechoic, anechoic, or hyperechoic regions. Unenhanced CT demonstrated mean attenuation values of 34 HU, and 6 tumors (15%) contained punctate calcification. Enhancement patterns on contrast-enhanced CT differed significantly based on tumor size (Table 3, Figs. 1 and 2). None showed local invasion or lymphadenopathy. Table 4 demonstrates the size-based differences in enhancement patterns of 16 patients with isolated AHs.

**Table 3.** Ultrasonography and contrast-enhanced computed tomography features of 40 adrenal hemangiomas

No.	Maximum tumor diameter (cm)	Echogenicity	Precontrast HU	Postcontrast HU	Enhancement feature	Calcification
1	3.7	/	NA^a^	NA	NA	No
2	3.9	/	46	52.6	Centripetal progressive	No
3	10.9	/	35.2	40.8	Centripetal progressive	Yes
4	1.7	Hypoanechoic	NA	NA	NA	No
5	3.3	/	29.8	33.5	Centripetal progressive	No
6	1.4	/	9.2	10.5	None	No
7	1.3	/	25.4	36.5	Mild	No
8	2.3	/	25.9	122.5	Marked	No
9	4.3	/	45.7	48.9	Centripetal progressive	Yes
10	7.2	/	33.6	37.5	Centripetal progressive	No
11	4.5	Hypoanechoic	32.6	43.0	Centripetal progressive	No
12	1.8	Medium-hyperechoic	58.8	68.4	Mild	No
13	1.4	Hypoechoic	NA	NA	NA	No
14	6.2	Hypoechoic	NA	NA	NA	No
15	1	/	NA	NA	NA	No
16	7.7	Hypoechoic	NA	NA	NA	No
17	2.1	/	33.2	94.2	Marked	No
18	4.3	Hypoechoic	NA	NA	NA	Yes
19	2.7	/	24.6	85.4	Marked	No
20	9.1	/	NA	NA	NA	No
21	4.3	Medium echoic	43.5	48.5	Centripetal progressive	Yes
22	3.4	/	NA	NA	NA	No
23	2.9	Hypoechoic	20.8	63.8	Moderate	No
24	1.4	/	35.3	42.0	Mild	No
25	2.2	/	29.2	55	Moderate	No
26	4.5	/	NA	NA	NA	No
27	3.2	/	NA	NA	NA	No
28	1.6	/	NA	NA	NA	No
29	4.1	Anechoic	NA	NA	NA	No
30	5.6	Anechoic	NA	NA	NA	No
31	5.5	Hypoanechoic	NA	NA	NA	No
32	2.5	/	NA	NA	NA	Yes
33	3.3	Hypoechoic	NA	NA	NA	No
34	4.8	Anechoic	NA	NA	NA	No
35	1.9	/	NA	NA	NA	No
36	8.3	Hypoanechoic	NA	NA	NA	No
37	4.8	/	40.9	45.2	Heterogenous#	No
38	3.2	/	NA	NA	NA	No
39	4	Hypoechoic	NA	NA	NA	Yes
40	10.6	Medium-hyperechoic	25.1	26.8	Centripetal progressive	No

Abbreviations: NA, not applicable.

**Table 4.** Size-based differences in enhancement patterns of 16 patients with isolated adrenal hemangiomas

	None to mild	Moderate to marked	Centripetal progressive	*P*-value
<2 cm	4	0	0	<.001
2 to 3 cm	0	5	0	<.001
>3 cm	0	0	7	<.001

### Treatment and Pathology

All 40 patients underwent adrenalectomy, with 39 (97.5%) undergoing laparoscopic adrenalectomy and 1 (2.5%) open surgery (Table 5). This included 2 simultaneous bilateral laparoscopic adrenalectomies (patients no. 11 and no. 22). Mean operative time was 96 minutes, and the mean estimated blood loss was 71 mL. All patients recovered well without complications.

**Table 5.** Surgery and pathology results of the 40 patients with AH

No.	Surgery approach	Tumor laterality	Surgical laterality	Pathology result
1	Laparoscopic	Left	Left	AH
2	Laparoscopic	Left	Left	AH
3	Laparoscopic	Left	Left	AH with bleeding
4	Laparoscopic	Right	Right	AH
5	Laparoscopic	Right	Right	AH
6	Laparoscopic	Left	Left	AH
7	Laparoscopic	Right	Right	AH with bleeding
8	Laparoscopic	Left	Left	AH
9	Laparoscopic	Right	Right	AH
10	Laparoscopic	Left	Left	AH
11	Laparoscopic	Bilateral	Bilateral	(left) adrenal adenoma; (right) AH with bleeding
12	Laparoscopic	Right	Right	AH
13	Laparoscopic	Right	Right	AH
14	Laparoscopic	Right	Right	AH
15	Laparoscopic	Left	Left	AH
16	Laparoscopic	Left	Left	AH with adrenal cortical hyperplasia
17	Laparoscopic	Left	Left	AH with adrenal cortical hyperplasia
18	Laparoscopic	Bilateral	Right^a^	AH with adrenal cortical hyperplasia
19	Laparoscopic	Right	Right	AH
20	Laparoscopic	Right	Right	AH with bleeding
21	Laparoscopic	Right	Right	AH
22	Laparoscopic	Bilateral	Bilateral	(left) adrenal adenoma; (right) AH
23	Laparoscopic	Right	Right	AH
24	Laparoscopic	Right	Right	AH
25	Laparoscopic	Left	Left	AH
26	Laparoscopic	Right	Right	AH
27	Laparoscopic	Right	Right	AH
28	Laparoscopic	Right	Right	AH
29	Laparoscopic	Right	Right	AH
30	Laparoscopic	Left	Left	AH
31	Laparoscopic	Right	Right	AH
32	Laparoscopic	Left	Left	AH
33	Laparoscopic	Left	Left	AH
34	Laparoscopic	Right	Right	AH
35	Laparoscopic	Left	Left	AH
36	Laparoscopic	Right	Right	AH with bleeding
37	Laparoscopic	Right	Right	AH with adrenal adenoma
38	Laparoscopic	Left	Left	AH with adrenal adenoma
39	Laparoscopic	Left	Left	AH with adrenal adenoma
40	Open	Right	Right	AH with pheochromocytoma

Abbreviations: AH, adrenal hemangioma.

**Figure 3.**
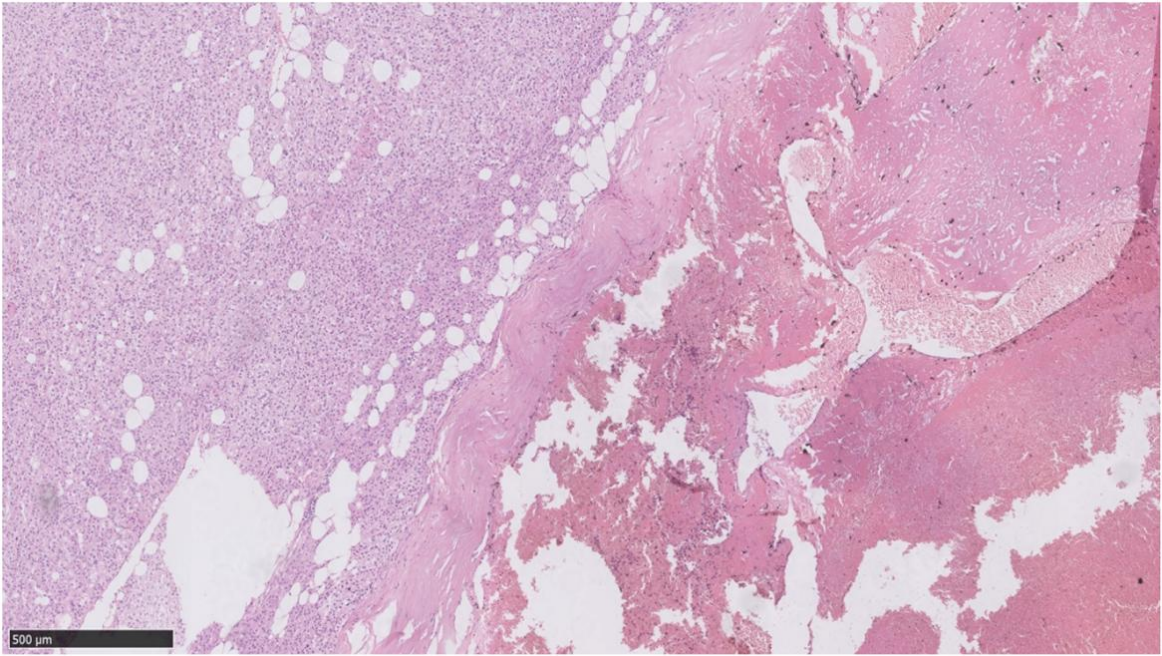
Photomicrographs showing histopathologic features of collision tumor of adrenal hemangioma with adrenal adenoma (hematoxylin and eosin stain, original magnification ×50). The left side shows adenoma and the right side shows hemangiomas with bleeding.

**Figure 4.**
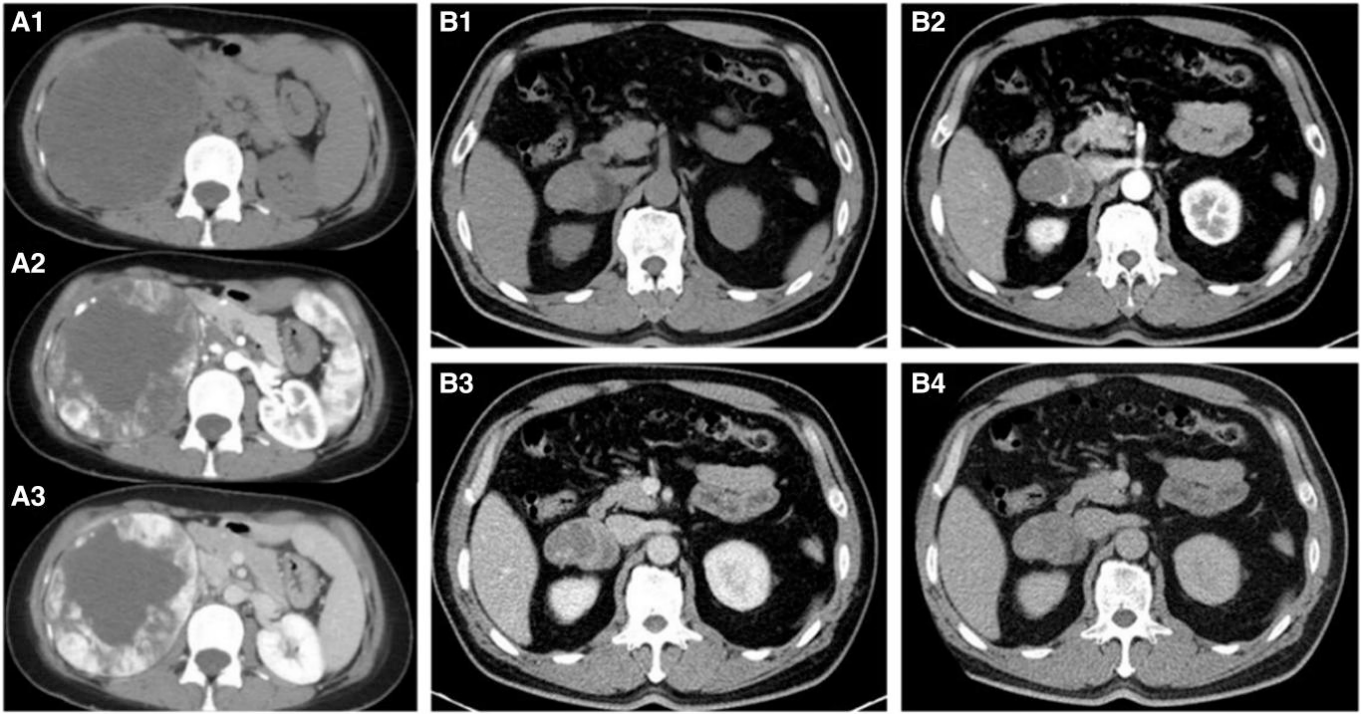
Collision tumors involving adrenal hemangioma. (A) Contrast-enhanced CT of a pheochromocytoma-hemangioma collision tumor. (A1-A3) shows typical centripetal progressive enhancement and central necrosis in the hemangioma combined with adrenal pheochromocytoma. (B) CT images demonstrating a hemangioma-adenoma collision tumor with distinct enhancement patterns. (B1) shows a higher density on the right side of the tumor on plain CT. (B2-B4) shows different enhancement features of the left and right sides of the tumor. The right side shows central progressive partial filling in the venous and delayed phase, while the left side shows moderate enhancement. Abbreviations: CT, computed tomography.

The pathology feature of AH is the composition of numerous congested, variably dilated vessels of different calibers. Positive immunohistochemical staining for CD34, CD31, and ERG may support the diagnosis of hemangioma. Pathological analysis identified 28 (70%) cases of simple AH. Five (12.5%) were AH with bleeding, with 1 patient having hypertension and palpitations and 1 patient having lower back pain. Three (7.5%) were AH with adrenal cortical hyperplasia. Four (10%) were adrenal collision tumors, including 3 AHs with adrenal adenoma (patients nos. 37 to 39) and 1 with pheochromocytoma (patient no. 40) (Figs. 3 and 4). Those with adenoma had mild cortisol [24-hour UFC 138.5 ug/24h (12.3-103.5)], or catecholamine [24-hour urine dopamine (487.9 ug/24h (0-459.9)] elevations without clinical features. The pheochromocytoma case presented with palpitation, and her 24-hour urine dopamine, norepinephrine, metanephrine, and 3-normetanephrine were significantly increased. Somatostatin receptor scintigraphy and MIBG were both positive. Therefore, she was diagnosed with pheochromocytoma preoperatively.

### Follow-up

All 40 cases are alive without relapse after a maximal follow-up of 15 years, and steroid replacement therapy was administered for 1 patient (no. 18). This patient presented with no symptoms of adrenal insufficiency preoperatively, however, with elevated ACTH (246 pg/mL), normal plasma cortisol, and normal 24-hour UFC. CT revealed bilateral adrenal tumors, with multiple larger masses up to 4.2 cm diameter on the right side demonstrating significant heterogeneous enhancement. A 1.3 × 2.1 cm nodule with heterogeneous enhancement was also noted on the left. Pathology of the right-side tumor showed AH combined with adrenal hyperplasia. Based on the pathology, we considered he could be diagnosed as AH combined with nonclassic congenital adrenal hyperplasia. Therefore, we did not perform the surgery on the left side tumor. The patient did not exhibit significant adrenal insufficiency postoperatively initially; however, symptoms emerged gradually during follow-up. Hydrocortisone was gradually increased from 10 mg once a day to 10 mg every 8 hours with a good response.

## Discussion

This study of 40 cases provides valuable insights into the clinical characteristics of AH. The 0.56% incidence we observed is higher than estimated, likely due to our large sample size. The peak incidence in ages 45 to 64 years and female predominance of 1.7:1 concur with previous reports. Most patients were asymptomatic and the AH was incidentally discovered during health examinations. However, 25% presented with difficult-to-control hypertension or related complications like headaches, palpitations, or hypokalemia. Sudden hypotension from rupture hemorrhage is another rare but potentially fatal presentation [3].

Most adrenal hemangiomas are hormonally inactive, but there are some rare cases reporting production of some adrenocortical hormones [5]. In 2 hypertensive hypokalemic cases, symptoms resolved after adrenalectomy, indicating that the tumors may produce mineralocorticoid hormone, though the mechanisms are unclear. Concomitant cortical hyperplasia is a possibility.

The tumor diameter of AH fluctuates greatly, and large tumors can reach over 30 cm [6]. They usually occur on 1 side but can also occur on both sides simultaneously [7]. Ultrasonography reveals variable echogenicity depending on cystic, solid, or mixed components. Nonspecific hypoechoic appearance contributes to misdiagnosis. Unenhanced CT shows heterogeneity, sometimes with punctate calcification or peripheral fat islands [8]. On MRI, hemangiomas are generally T1 hyper- or hypointense and T2 hyperintense. On enhanced CT or MRI, hemangiomas typically demonstrate early peripheral nodular enhancement in the arterial phase, with centripetal progressive partial filling in the venous and delayed phase [9], because of the blood-filled sinusoids located at the edge of the tumor. Our analysis revealed size-based differences in enhancement patterns likely related to vascular distribution. Tumors <2 cm exhibit only none to mild enhancement, resembling adenomas. Tumors 2 to 3 cm show marked peripheral enhancement, resembling pheochromocytomas. Tumors >3 cm demonstrate the classic centripetal progressive filling in and central necrosis.

The radiological characteristics of different rare benign adrenal lesions may vary from distinct to indeterminate. Adrenal cortical adenoma is usually well circumscribed and homogeneous on nonenhanced images, accompanied by homogeneous contrast material enhancement. The attenuation values of adenomas rely on the quantity of lipids. Generally, a lipid-rich adenoma is diagnosed when an adrenal lesion measures less than 10 HU on nonenhanced CT images. Lipid-poor adenomas constitute about 10% to 40% of adenomas and exhibit higher attenuation on nonenhanced CT images [10, 11]. Since the first description of adrenal washout CT in a 1998 CT [12], this method has been reported repeatedly for the diagnosis of benign or malignant adrenal lesions. In short, the idea is that the percentage washout of contrast agent after its 10-to 15-minute application is high in benign adrenal lesions and low in malignant adrenal lesions. Threshold values of more than 60% for absolute contrast material washout and 40% for relative contrast material washout are highly sensitive and specific for diagnosing adrenal adenomas [13]. In a recent population-based study on adrenal tumors [14], the attenuation value of pheochromocytomas was 33 HU and no tumor had an attenuation value less than 10 HU. The necessity of biochemical evaluation for adrenal incidentals with attenuation values less than 10 HU has been discussed as evidence for benign adrenal adenomas, but clear evidence is lacking. Phaeochromocytoma should be included with adenomas in the differential diagnosis both for masses with low attenuation on nonenhanced CT and for lesions exhibiting a high percentage of contrast washouts. Gross features of phaochromocytomas described in the radiology literature are cystic regions, calcifications, fibrosis, necrosis, and internal hemorrhage [15]. Angiomyolipoma is a rare benign mesenchymal tumor composed of mature adipose tissue, smooth muscle, and blood vessels. The appearance of angiomyolipoma is indistinguishable from that of adrenal myelolipoma. Due to the diverse morphology of angiomyolipoma on imaging, it is difficult to confirm the diagnosis, and if the tumor is large and heterogeneous, imaging may give a false impression of malignancy [16, 17].

The surgical intervention for pheochromocytoma is associated with a high risk of hemodynamic instability. To mitigate the potential complications during the perioperative phase, adequate preoperative alpha blockade treatment aimed at normalizing blood pressure and restoring blood volume is necessary and required. In our center, for the medical safety of patients, if there are suspected signs of pheochromocytoma on imaging before surgery, even if other tests are negative, we would still prepare medication and perform surgery according to pheochromocytoma because inadequately prepared pheochromocytoma would lead to severe blood pressure fluctuations during surgery. Thus, when pheochromocytoma cannot be clinically ruled out, we prefer preoperative preparation for it regardless. However, our study concludes that for a tumor over 3 cm exhibiting the typical centripetal enhancement and central necrosis without typical symptoms or catecholamine elevation, AH should be suspected instead to avoid unnecessary medication. Distinguishing a 2 to 3 cm tumor by imaging alone proves difficult, necessitating a combination of clinical symptoms, endocrine tests, somatostatin receptor scintigraphy, and MIBG to determine the diagnosis and preoperative preparation needs.

Adrenal collision tumors are defined as the coexistence of 2 adjacent but histologically distinct tumors of the adrenal gland without a substantial histologic admixture at the interface. Various types of adrenal collision tumors have been reported, such as adrenal adenoma and pheochromocytoma [18], adrenal adenoma and hemangioma [19], adrenal adenoma and metastatic carcinoma [20], adrenal cortical carcinoma and metastatic carcinoma [21], etc. Due to the different components within tumors, their endocrine functions are uncertain. Treatment should be determined by the functional parts. AH usually occurs in the adrenal cortex, and the combination with pheochromocytoma is extremely rare. Preoperative preparation and treatment methods should be conducted as the pheochromocytoma.

Despite the generally good prognosis of AH, some patients still experience severe complications from bleeding or compression by a large tumor. The preferred treatment is surgical resection. Laparoscopic adrenalectomy is recommended for tumors under 6 cm [22]. However, improved laparoscopic skills, especially robot-assisted approaches, make tumor size less significant in choosing the surgical method. In our study, 6 patients with tumors over 6 cm, up to 10.9 cm, underwent laparoscopic surgery and recovered well without major perioperative complications.

Due to the retrospective nature of this study, some clinical data such as imaging findings and follow-up information were incomplete. This limited our ability to fully evaluate the postoperative recovery of hypertension and its impacts on adrenal cortical function.

## Conclusion

This large cohort study provides valuable insights into AH clinical characteristics including its demographics, diagnostic challenges, and imaging features, revealing distinct size-based enhancement patterns on CT. Recognizing imaging features can improve diagnostic accuracy. Laparoscopic adrenalectomy is the preferred treatment. Further research is warranted to enhance the recognition of this rare tumor.

## Data Availability

Some or all datasets generated during and/or analyzed during the current study are not publicly available but are available from the corresponding author on reasonable request.
